# Treatment outcomes of drug resistant tuberculosis patients in Morocco: multi-centric prospective study

**DOI:** 10.1186/s12879-019-3931-5

**Published:** 2019-04-11

**Authors:** Mariam El Hamdouni, Jamal Eddine Bourkadi, Jouda Benamor, Mohammed Hassar, Yahia Cherrah, Samir Ahid

**Affiliations:** 1 Equipe de Recherche de Pharmacoéconomie & Pharmacoépidémiologie. Laboratoire de Pharmacologie & Toxicologie, Faculté de Médecine et de Pharmacie, Université Mohammed V, Rabat, Morocco; 2 Service de Pneumologie, Hôpital My Youssef, Rabat, Morocco

**Keywords:** Resistant tuberculosis, Anti-tuberculosis drugs, Treatment outcomes, Morocco

## Abstract

**Background:**

Drug resistant tuberculosis is a major public health problem in Morocco and worldwide. Treatment outcome of drug resistant tuberculosis is poor and requires a long period of treatment with many toxic and expensive antituberculosis drugs. The aim of this study is to evaluate treatment outcomes of drug resistant tuberculosis and to determine predictors of poor treatment outcomes in a large region of Morocco.

**Methods:**

It is a multi-centric observational cohort study conducted from January 01, 2014 to January 01, 2016. A questionnaire was established to collect data from clinical charts of patients with confirmed resistant TB. The study was carried out in all the 11 centers located in the Rabat-Salé-Kénitra region of Morocco where drug resistant tuberculosis is treated. Treatment outcomes were reported and the definitions and classifications of these outcomes were defined according to the WHO guidelines. Univariate and multivariate logistic regression were conducted to determine factors associated with poor drug resistant tuberculosis treatment outcomes in Morocco.

**Results:**

In our study, 101 patients were treated for drug resistant tuberculosis between January 01, 2014 and January 01, 2016. Patients’ age ranged from 9.5 to70 years; 72patients (71.3%) were male and 80 patients (79.2%) were living in urban areas. Thirty two patients were smokers, 74 patients had multidrug-resistant tuberculosis, 25 patients had rifampicin resistance and 2 patients had isoniazid resistance. Treatment outcomes of tuberculosis patients were as follows: 45 patients were cured (44.5%), 9 completed treatment (8.9%), 5 patients died before completing the treatment, 35 patients were lost to follow up (34.6%) and 7 patients had treatment failure. In the multivariate analysis, being a smoker is an independent risk factor for poor treatment outcomes, (*p*-value = 0.015, OR = 4.355, IC [1.327–14.292]).

**Conclusion:**

Treatment success outcomes occurred in more than half of the cases, which is lower than the World Health Organization target of at least a 75% success rate. A significant number of patients abandoned their treatment before its completion. These dropouts are a serious public health hazard that needs to be addressed urgently.

## Background

Tuberculosis (TB) remains a serious global public health problem in Morocco and worldwide. It is caused by *Mycobacterium tuberculosis* that mostly affects the lungs. In 2016, the World Health Organization (WHO) estimated that there were 10.4 million persons infected by *Mycobacterium tuberculosis*, equivalent to 140 cases per 100,000 inhabitants and 1.7 million deaths caused by TB [1].

MDR-TB is defined as resistance to isoniazid and rifampicin, with or without resistance to other first-line anti-TB drugs. XDR-TB is defined as resistance to isoniazid and rifampicin, and to any fluoroquinolone, and to any of the 3 second-line injectable medicines (amikacin, capreomycin, and kanamycin). Rifampicin resistance (RR) is defined as resistance to rifampicin with or without resistance to other anti-TB drugs [2].

Multidrug-resistant TB (MDR-TB) is multifactorial and is fuelled by improper treatment of sensitive TB patients, premature TB treatment interruption, and airborne transmission of resistant bacteria in public places [3]. In 2016, an estimated 600,000 new cases of MDR-TB and RR-TB were registered as well as 240,000 deaths caused by MDR/RR and 8000 cases of extensively drug-resistant tuberculosis (XDR-TB) [1].

In Morocco, 36, 000 cases of TB were notified in 2016, an incidence of 103 cases per 100,000 inhabitants; 3300 deaths were caused by TB and 160 patients developed MDR-TB in 2015 [4].

The treatment of resistant TB is complex, long, expensive. It requires many antituberculosis drugs that are more toxic and less effective than the first line medicines administered for sensitive TB; this often results in poor treatment outcomes [5]. In one study, only 54 of the MDR/RR-TB patients who started the treatment in 2014 were successfully treated, 16% of patients died, 8% of patients had treatment failure. Among XDR-TB patients, the success rate was 30% [6–8].

Many studies have been conducted in several countries evaluating treatment outcomes of drug resistant tuberculosis (DR-TB) patients and yielded different results [9–12]. However, no study has assessed treatment outcomes of DR-TB and factors associated with poor treatment outcomes in Morocco. The purpose of this study is to evaluate treatment outcomes of DR-TB patients and to determine factors associated with poor treatment outcomes prospectively in one of Morocco largest region.

## Methods

### Study setting

Our study setting is the Rabat-Salé-Kénitra region, one of the twelve administrative regions of Morocco. Located in the North-Western part of Morocco, it has an area of 17,570 km2 and a population of 4,580,866 inhabitants. It is Morocco’s second most populated region with 13.5% of the national population. It is also the second region for TB notifications in 2015 (17.1% of all cases) [13]. This is a multi-centric observational cohort study that lasted twenty four months, from January 01, 2014 to January 01, 2016. This study was carried out in all the 11 centers of the Rabat-Salé-Kénitra region where resistant TB is treated. They are located in the cities of Rabat, Salé, Témara, Kénitra, Khémisset and Sidi kacem. A small number of patients have suspected XDR-TB, and were therefore excluded from the study.

### Diagnosis and DR-TB treatment

All patients diagnosed with resistant TB during the study were included. The diagnosis was done by Xpert MTB/RIF and drug susceptibility testing (DST) for first-line anti-TB drugs (rifampicin, isoniazid, streptomycin and ethambutol) and second-line anti-TB drugs (ofloxacin, kanamycin and capreomycin). For Xpert MTB/RIF, one sample is always collected before starting treatment. DST method is based on solid culture (Löwenstein-Jensen). DST critical concentrations of the anti-tuberculosis drugs are: isoniazid: 0.2 μg/ml; rifampicin: 40 μg/ml; ethambutol: 2 μg/ml; streptomycin: 4 μg/ml; kanamycin: 30 μg/ml; capréomycin: 40 μg/ml; ofloxacin: 2 μg/ml [14]. All DR-TB patients were tested for HIV at baseline.

DR-TB regimen consisted of an intensive phase lasting a minimum of 6 months and consisting of an injectable agent (kanamycin or capreomycin), in addition to levofloxacin, ethionamide, cycloserine, pyrazinamide, ethambutol and vitamin B6. It was then followed by the continuation phase during 18 months and consisting of the regimen prescribed for the intensive phase without the injectable agents. The duration of DR-TB treatment was 24 months. All drugs were administrated under directly observed therapy (DOT).

Sputum smears and cultures were obtained monthly during the intensive phase of DR-TB treatment. Second-line DST was performed at baseline and was redone at 3 and 6 months if culture results remained positive. During the continuation phase, sputum smears and cultures were obtained every 3 months, and second-line DST is redone for all positive cultures.

### Data collection

A data collection questionnaire was designed using the medical charts of all the patients diagnosed with resistant TB during the study period. DR-TB was confirmed by drug susceptibility testing (DST) for first-line anti-TB medicines and for second-line medicines. Patients included received second-line anti-TB medicines including ethionamide, kanamycin or capreomycin, cycloserine and ofloxacin. The duration of treatment was 24 months. All socio-demographic and clinical data were prospectively collected: age, gender, residence, marital status, employment, comorbidity, TB drug resistance types, smoking habits, culture positivity, smear positive pulmonary tuberculosis (PTB+) at baseline, adverse reactions (ADR) and treatment outcomes. ADRs and their dates of onset were obtained from the patients’ charts. Cytolysis or hepatic cytolysis is defined as liver transaminases [Aspartate aminotransferase (ASAT) and Alanine aminotransferase (ALAT)] higher than the upper normal limit in the presence of symptoms such as nausea, anorexia, vomiting, or abdominal pain. It was assessed by blood liver function tests.

### Treatment outcomes

Treatment outcomes were defined and classified according to the WHO guidelines [15].

Cured from DR-TB was defined as those who completed treatment within 18 months to over 2 years, followed by at least two negative sputum cultures.

Completed treatment was defined as patients who completed the anti TB regimen for at least 18 months.

Death was defined as patients who died during treatment whatever the cause.

- Failed treatment was defined as smear positive patients who remained positive at the fifth month of treatment or smear negative turning positive.
- Lost to follow-up was defined as treated patients who did not come back to complete chemotherapy and there was no evidence of cure through the sputum result during the fifth month of therapy. Treatment interruption was defined as patients who did not collect medications for 2months or more at a particular time or only intermittently, but still came back for treatment and in the 8th month of treatment their sputum result was positive.
- TB relapse refers to patients who had previous TB treatment and were cured but were diagnosed again with a new TB infection.
- Successful treatment outcomes include cured patients and those who completed treatment. Poor treatment outcomes include death, TB relapse, loss to follow-up and failure to complete treatment regimen [8].

### Statistical analysis

The data were analyzed by SPSS 13.0 software. Quantitative variables were expressed by means and standard deviations (m ± SD), qualitative variables were expressed by frequencies (n) and percentages (%). We conducted univariate and multivariate binary logistic regressions (entry method) to determine factors associated with poor DR-TB treatment outcomes. Known risk factors of poor TB treatment outcomes variables such as age, gender, residence, employment status, previous TB history, PTB+ at baseline, smoking status and presence of comorbidity are included into univariate and multiple logistic regression analysis in-order to predict the final independent factors. The significance of the statistical tests was taken at a *p*-value of < 0.05.

### Ethics statement

The study was approved by the Ministry of Health of Morocco and by the Ethics Committee of the Faculty of Medicine and Pharmacy, Rabat under the reference number 706/2014. All patients’ data were rendered anonymous before analysis.

## Results

### Socio-demographic and clinical characteristics

A total of 101 patients were treated for DR-TB. The patient s’ mean age is 35.5 ± 13.3 years, ranging from 9.5 to 70 years. The majority of patients were male (*n* = 72, 71.3%); 50 patients were unemployed (49.5%) and 80 patients resided in urban areas (79.2%). According to TB drug resistance types, 74 patients had MDR-TB; they were divided into 41 patients who had resistance to isoniazid & rifampicin and 33 patients who had in addition to isoniazid & rifampicin resistance, other anti-TB drug resistance. Twenty five patients had rifampicin resistance and 2 patients had isoniazid resistance. Twenty patients had comorbidity and 32 were smokers. We found also that 83.2% of patients were previously treated for TB, 81.2% were PTB+ at baseline and 46 had a positive culture during the intensive phase. Identified clinical and socio-demographic characteristics of DR-TB patients are reported in Table 1.

**Table 1:** Socio-demographic and clinical characteristics of DR-TB patients in the Rabat-Salé-Kénitra region, from to 2014 to 2016

Characteristics	n (%)
Total of patients	101 (100)
Age (m ± SD)	35.5 ± 13.3
Age group years	
<=40	64 (63.4)
>40	37 (36.6)
Gender	
Male/female	72/29 (71.3/28.7)
Residence	
Urban/rural	80/21 (79.2/20.8)
Employment	
Unemployed	50 (49.5)
Employed	24 (23.8)
Not available	27 (26.7)
Marital status	
Single/married	41/45 (40.6/44.5)
Not available	15 (14.8)
Previous TB history	84 (83.2)
Characteristics	**n (%)**
PTB+ at baseline	82 (81.2)
Smoking habits	32 (31.7)
Comorbidity	20 (19.8)
Culture positivity during intensive phase TB drug resistance types	46 (45.5)
MDR	74 (73.3)
INH + RIF	41 (40.6)
INH + RIF + STP	17 (16.8)
INH + RIF + STP + MTB	9 (8.9)
INH + RIF + MTB	4 (4)
INH + RIF + MTB + STP + Ofx	1 (1)
INH + RIF + STP + PZA	1 (1)
INH + RIF + Eto + Km	1 (1)
Rifampicin resistance	25 (24.7)
Isoniazid resistance	2 (2)
ADR	49 (48.5)

*m* mean, *SD* standard deviation, *TB* tuberculosis, *PTB*+ smear positive pulmonary tuberculosis, *MDR* multidrug-resistant tuberculosis, *INH* Isoniazid, *RIF* Rifampicin, *PZA* Pyrazinamid, *MTB* Ethambutol, *STP* Streptomycin, *Ofx* Ofloxacin; Eto: Ethionamide, *Km* Kanamycin, *ADR* adverse reactions

### Adverse reactions

Among the 101 DR-TB patients included in the study, 49 had adverse drug reactions constituting a total of 98 events; 94% of them occurred in the intensive phase of treatment. The majority were common adverse events, mainly gastrointestinal (vomiting, anorexia, abdominal pain, diarrhea, nausea) (*n* = 43, 42.6%). Pruritus and or rash were noted in 12 patients (11.9%), 7 patients (6.9%) complained of paresthesia and joint pain and 2 patients (2%) had polyarthralgia. Eight patients had hearing impairment (7.9%). Cytolysis and jaundice occurred in 2 patients (2%). Neurologic adverse reactions were observed in 16 patients (15.8%) and psychic troubles in 2 patients (2%). Three patients (3%) had edema. Two patients (2%) had fever and one patient (1%) had thyroid hypertrophy.

### Treatment outcomes

Treatment outcomes of our DR-TB patients were as follows: 54 had successful treatment (53.5%), 45 were cured (44.5%) and 9 completed treatment (8.9%). Poor treatment outcome was seen in 47 patients (46.5%), 5 patients died before completing treatment, 7 patients had treatment failure and 35 patients were lost to follow-up (34.6%). Patients who were lost to follow-up interrupted treatment after a median of 62 days (IQR 0–204 days). Most of these interruptions occurred during the intensive phase of treatment (77%). Treatment outcomes by resistance pattern across the different centers of Rabat-Salé-Kénitra region are summarized in Tables 2 and 3.

**Table 2:** Treatment outcomes of DR-TB patients in the Rabat-Salé-Kénitra region by resistance pattern, from to 2014 to 2016

Treatment outcomes, n (%)	MDR	Rifampicin resistance	Isoniazid resistance	Total
Cured	34 (33.7)	10 (9.9)	1 (1)	45 (44.5)
Completed	7 (6.9)	2 (2)	0 (0)	9 (8.9)
Lost to follow up	23 (22.8)	11 (10.9)	1 (1)	35 (34.6)
Failed	7 (6.9)	0 (0)	0 (0)	7 (6.9)
Died	3 (3)	2 (2)	0 (0)	5 (4.9)
Successful treatment (cured + completed)	41 (40.6)	12 (11.9)	1 (1)	54 (53.5)
Poor treatment (failure + death + lost to follow up)	33 (32.7)	13 (12.9)	1 (1)	47 (46.5)

*MDR* multidrug-resistant tuberculosis

**Table 3:** Treatment outcomes of DR-TB patients in the Rabat-Salé-Kénitra region across different centers, from to 2014 to 2016

Treatment outcomes, n (%)	Salé (1 center)	Khémisset (1 center)	Témara (1 center)	Rabat (6 centers)	Kénitra (1 center)	Sidi kacem (1 center)	Total
Cured	16 (15.8)	8 (7.9)	9 (8.9)	3 (3)	3 (3)	6 (5.9)	45 (44.5)
Completed	3 (3)	5 (4.9)	0 (0)	1 (1)	0 (0)	0 (0)	9 (8.9)
Lost to follow up	11 (10.9)	2 (2)	4 (4)	8 (7.9)	8 (7.9)	2 (2)	35 (34.6)
Failed	1 (1)	2 (2)	2 (2)	1 (1)	1 (1)	0 (0)	7 (6.9)
Died	3 (3)	1 (1)	1 (1)	0 (0)	0 (0)	0 (0)	5 (4.9)
Successful treatment (cured + completed)	19 (18.8)	13 (12.9)	9 (8.9)	4 (4)	3 (3)	6 (5.9)	54 (53.5)
Poor treatment (failure + death + lost to follow up)	15 (14.8)	5 (4.9)	7 (6.9)	9 (8.9)	9 (8.9)	2 (2)	47 (46.5)
Total	34 (33.7)	18 (17.8)	16 (15.8)	13 (12.9)	12 (11.9)	8 (7.9)	101 (100)

### Predictors of poor treatment outcomes

In the univariate analysis, poor treatment outcomes were associated with male gender (*p*-value =0.006, OR = 3.929, IC [1.490–10.356]) and being a smoker (*p*-value < 0.001, OR = 6.00, IC [2.335–15.418]). After adjusting for the effects of potential confounders in the multivariate regression analysis, being a smoker remained an independent risk factor for poor treatment outcomes, (*p*-value = 0.015, OR = 4.355, IC [1.327–14.292]). The univariate and multivariate analysis are in Table 4.

**Table 4:** Predictors of poor treatment outcomes: simple and multiple logistic regression analysis

Independent variable	Treatment outcomes, n (%)		Univariate analysis			Multivariate analysis		
	Poor treatment(*n* = 47)	Successful treatment(*n* = 54)	*P* value	OR	95% CI	*P* value	Adjusted OR	95% CI
Age < =40 years								
No	20 (54.1)	17 (45.9)		1			1	
Yes	27 (42.2)	37 (57.8)	0.251	0.620	[0.275–1.401]	0.535	0.704	[0.232–2.136]
Gender								
Female	7 (24.1)	22 (75.9)		1			1	
Male	40 (55.6)	32 (44.4)	**0.006**	3.929	[1.490–10.356]	0.231	2.215	[0.602–8.151]
Residence								
Rural	8 (38.1)	13 (61.9)		1			1	
Urban	39 (48.8)	41 (51.2)	0.386	1.546	[0.578–4.134]	0.350	1.943	[0.483–7.820]
Employment								
Yes	13 (54.2)	11 (45.8)		1			1	
No	23 (46.0)	27 (54.0)	0.511	0.721	[0.271–1.914]	0.388	1.753	[0.491–6.263]
PTB+ at baseline								
No	8 (42.1)	11 (57.9)		1			1	[0.327-
Yes	39 (47.6)	43 (52.4)	0.668	1.247	[0.455–3.419]	0.599	1.506	6.937]
Previous TB history	7 (41.2)	10 (58.8)		1			1	
No	40 (47.6)	44 (52.4)	0.628	1.299	[0.452–3.736]	0.671	0.707	[0.143–3.501]
Yes								
Smoking habits								
No	23 (33.3)	46 (66.7)		1			1	
Yes	24 (75.0)	8 (25.0)	**< 0.001**	6.00	[2.335–15.418]	**0.015**	4.355	[1.327–14. 292]
Comorbidity								
No	40 (49.4)	41 (50.6)		1			1	
Yes	7 (35.0)	13 (65.0)	0.252	0.552	[0.200–1.526]	0.902	1.090	[0.277–4.293]

*In bold: p* value less than 0.05, TB: Tuberculosis; PTB+: smear positive pulmonary tuberculosis; OR: odds ratio; CI: confidence interval;1:reference

## Discussion

This is the first study in Morocco that evaluates treatment outcomes of DR-TB and predictors of poor DR-TB treatment outcomes. Of the 101 DR-TB patients in this study only 54 (53.5%) were successfully treated; these outcomes were lower than the 2015 WHO target for MDR TB of at least 75% treatment success [16].

In our study, the majority of patients were males (71.3%) which is in accordance with other studies that found that DR-TB is more common in males [17–20]. Most of our patients are young, with a mean age of 35.5 years, in agreement with the study of Selim et al who found a mean age of 39.35 years [21]. Unemployment was noted in 49.5% of the patients, which is in accordance with the study of Marta Gomes et al who found 51.8% were unemployed patients [22]. DR-TB is frequently seen in patients who have low socioeconomic status and who are illiterate and unaware of the risks to others as well as to themselves.

According to TB drug resistance types, the majority of patients were MDR-TB. This is in agreement with Meressa D et al who found 76.1% of patients with DR-TB were MDR-TB (270/355) [23].

Successful treatment outcomes in our study were slightly lower than those found in studies done in Shanghai, New York and Hamburg, (54.9, 64 and 80% respectively) [24–26]. But, they were higher than those found in studies done in Ukraine, South Korea and South Africa (18, 48.2 and 49% respectively) [9, 27, 28]. The success rate in our study was higher than the one found by Elmi et al [29] and Kim et al [30] who found a success rate of 17.1 and 39% respectively.

One of the most serious obstacles for TB control efforts is the low rate of treatment success among DR-TB patients as this might lead to the development of more resistance and the transmission of these more resistant strains to other persons. The major factor hindering treatment success is the high number of patients who are lost to follow-up [31]. In our study, we found 34.6%, which was a high loss to follow-up rate. This rate was higher than in studies done in Pakistan, Spain, South Africa and South Korea which found a loss to follow- up rate of 1.1, 16, 29 and 32% respectively [9, 32–34]. But it was lower than the 55.6% found by Rao NA et al. Study done in Karachi, (322 out of 579 were lost to follow-up) [35]. Loss to follow up occurred during the intensive phase of treatment. In a study done in Ethiopia, it was also shown that loss to follow-up occurred during the intensive phase of treatment [36]. However, a Chinese study found that loss to follow up occurred during the continuation phase of DR-TB treatment [12]. A recent systematic review and meta-analysis including 75 studies reported a mean loss to follow-up rate of 14.8% (12.4–17.4%). These studies identified the use of community health workers and DOT during treatment as strategies related to lower loss to follow-up rates [31]. Among possible reasons for loss to follow-up or discontinuation of therapy in our study were the adverse drug reactions, the long duration of treatment and an improvement in symptoms. The study of Holtz et al. [37] highlighted lack of patient-provider interaction, drug use, and socioeconomic characteristics as the most significant factors associated with loss to follow-up.

In our study, the failure rate of 6.9% was slightly lower than the 8 and 8.7% failure rates found in 2 other studies of DR-TB patients [38, 39]. The mortality rate was similar to the findings of Shin et al. who found 5% of death among MDR-TB patients [40].

In our study, smoking habits were associated with poor treatment outcomes, These results are in accordance with the results of the study of Tachfouti et al. [10] and a study conducted by Albuquerque et al. [41], they evaluated the association of smoking and unsuccessful treatment outcomes among TB patients. Another study by Bastos et al. reported that treatment success was more likely in non-smokers patients [17]. Smoking has been found to be associated with both relapse of TB and TB mortality [42].

Previous studies showed that baseline ofloxacin resistance was associated with poor MDR-TB treatment outcomes [12, 43, 44]. A study by Aibana et al. found other predictors of poor outcomes such as HIV infection without anti-retroviral therapy and presence of extensively-drug resistant TB while other variables were known as predictors of poor outcomes such as history of TB treatment, smear positivity and unemployment [9]. That was not the case in our study.

## Conclusion

Our study is the first study to evaluate treatment outcomes of DR-T in a large region of Morocco. Our study has its limitations. It only relied on patients’ charts, so we could not investigate directly these patients for additional variables and for many patients we did not have information about culture results during the intensive phase of their treatment. Nonetheless, we can conclude that the unsuccessful treatment outcome rate was high in our study. This is due to the high rate of patients who interrupted on their treatment before its completion. These results highlight the seriousness DR-TB in our region. Many efforts and means should be devoted to try to decrease the number of loss to follow-up.

## Abbreviations

ADR: adverse reactions
anti-TB drugs: antituberculosis drugs
DOT: directly observed therapy
DR-TB: drug resistant tuberculosis
DST: drug susceptibility testing
Eto: ethionamide
INH: isoniazid
Km: kanamycin
MDR-TB: multidrug-resistant Tuberculosis
MTB: ethambutol
Ofx: ofloxacin
PTB+: smear positive pulmonary tuberculosis
RIF: rifampicin
RR-TB: rifampicin-resistant Tuberculosis
STP: streptomycin
TB: tuberculosis
WHO: world health organization
XDR-TB: extensively drug-resistant tuberculosis
